# Virtual Reality Therapy as an Adjunct to Physiotherapy for Postoperative Pain Management: A Case Report of Laparoscopic Open Pyeloplasty for Hydronephrosis

**DOI:** 10.7759/cureus.48254

**Published:** 2023-11-04

**Authors:** Saurabh N Puri, Swapna Jawade, Nikita S Deshmukh

**Affiliations:** 1 Department of Musculoskeletal Physiotherapy, Ravi Nair Physiotherapy College, Datta Meghe Institute of Higher Education and Research, Wardha, IND

**Keywords:** virtual reality-based rehabilitation, patient outcomes, pain management, breathing exercises, postoperative care, rehabilitation, hydronephrosis, abdominal surgery, physiotherapy

## Abstract

Hydronephrosis occurs when the tubes connecting the kidneys and bladder become blocked. These tubes carry urine from the kidneys, where it is created, to the bladder, where it is stored until elimination. When one or both ureters get blocked, serious complications and symptoms might arise. These symptoms include urinary tract obstruction, urine backflow, kidney distension, increased intra-renal pressure, impaired kidney function, infection risk, urinary incontinence, which is the involuntary loss of urine, and discomfort in the side, abdomen, or groin. This case report describes the effective postoperative management of abdominal surgery with virtual reality therapy (VRT) combined with standard medical care as additional therapy and physical therapy for hydronephrosis in a 22-year-old male patient. After undergoing laparoscopic open pyeloplasty, the patient developed right-sided hydronephrosis due to ureteral stenosis. He had a ureteral stent inserted and received physical therapy, including pelvic floor muscle training, core strengthening, and diaphragmatic breathing exercises. After four weeks of physical therapy, the patient reported improvement in his symptoms, including reduced discomfort and increased urination. These findings imply that physical therapy, in addition to advanced treatment with the help of VRT for hydronephrosis following abdominal surgery, might be beneficial.

## Introduction

Hydronephrosis is defined as a condition characterized by having more than 1 liter of urine or 1.6% of body weight in the collecting system [1]. Hydronephrosis develops gradually and in most cases, less than 2 liters of body fluid (urine) is collected. The most common symptom in these patients is kidney stone disease. From an epidemiological standpoint, the left kidney is the most commonly afflicted organ. Urogenital junction (UPJ) blockage and kidney stones are the most prevalent underlying causes. Most cases of this disease are discovered in infancy and early childhood (congenital). Continued inflammation can cause progressive and progressive sequelae, such as hypertension, renal failure, and malignancy. If not treated promptly, it can become malignant [2]. The most frequently reported causes of hydronephrosis include bladder outlet obstruction, pelvic ureteric junction (PUJ) stenosis, kidney or ureteric calculi, pregnancy, and stenosis of the PUJ [3]. Ultrasound (US) imaging is a popular, widely accessible, noninvasive, and radiation-free imaging technology, and it is very helpful in the grading and diagnosis of hydronephrosis [4].

Abdominal procedures are performed on millions of patients worldwide each year, making them a common treatment method. Even though these operations are regularly required, they may have a detrimental impact on a patient's health. Patients may feel anxiety, sadness, and other negative psychological symptoms following surgery [5]. In this report, we present a rare case of a 22-year-old male patient in whom hydronephrosis due to UPJ obstruction was diagnosed and treated, and physical rehabilitation was performed along with the help of virtual reality therapy (VRT) to bring him back to the premorbid state. VR has been used for years to distract patients from their agony during painful medical procedures [6]. The use of VR immersion as an alternative to intravenous (IV) sedation during endoscopic urologic surgery under spinal anesthesia is a game-changing advancement in the field of surgical operations. This novel strategy has been proven to outperform standard sedation procedures in a variety of ways, providing numerous benefits to both patients and medical practitioners. Some major advantages of VR immersion over IV sedation in this context include enhanced patient comfort, lower cognitive distraction, improved intraoperative communication, tailored pain management, and better patient satisfaction [7].

A recent study found that the use of VR settings has the potential to improve the efficiency of regulated breathing methods, which are well known for their stress and anxiety-reduction effects. This case presentation focuses on critical outcome indicators and delves into parameters such as baseline mobility, pain management, and respiratory function.

## Case presentation

Patient information

A 22-year-old male patient presented in August 2022 with a history of trauma due to intense activity, which had led to pain in the upper back region and difficulty in activities of daily living. He had visited a physician who advised some investigations for the same, which confirmed hydronephrosis in the right kidney, which was an incidental finding. Hence, ultrasonography (USG) of the abdomen and pelvis was performed, which also showed gross hydronephrosis in the right kidney. A DTPA scan for further investigation showed obstructive delay at the PUJ level with hydronephrosis in the right kidney. After examination, he was diagnosed with PUJ obstruction with hydronephrosis and recommended admission to the surgical intensive care unit (SICU). He underwent laparoscopy-open pyeloplasty under general anesthesia on January 18, 2023, with a transverse incision over the abdomen.

Clinical findings

On examination, a Foley catheter and drainage tube were located in the right lower abdomen. During the examination, chest wall motion was reduced with the involvement of respiratory support muscles. Also, the patient's cardiovascular parameters were confirmed to be normal, his pulse was 90 beats per minute, and his blood pressure was 150/90 mmHg. Respiration was normal, abdominothoracic, and the respiratory rate was 20 per minute, and SPO_2_ was 99%. Auscultation revealed decreased air intake in both regions of the lungs. The patient manifested respiratory muscle weakness and early fatigue in activities of daily living. On postoperative day one, he was placed in the supine position with adequate back support for clinical examination.

Diagnostic assessment

**Figure 1:**
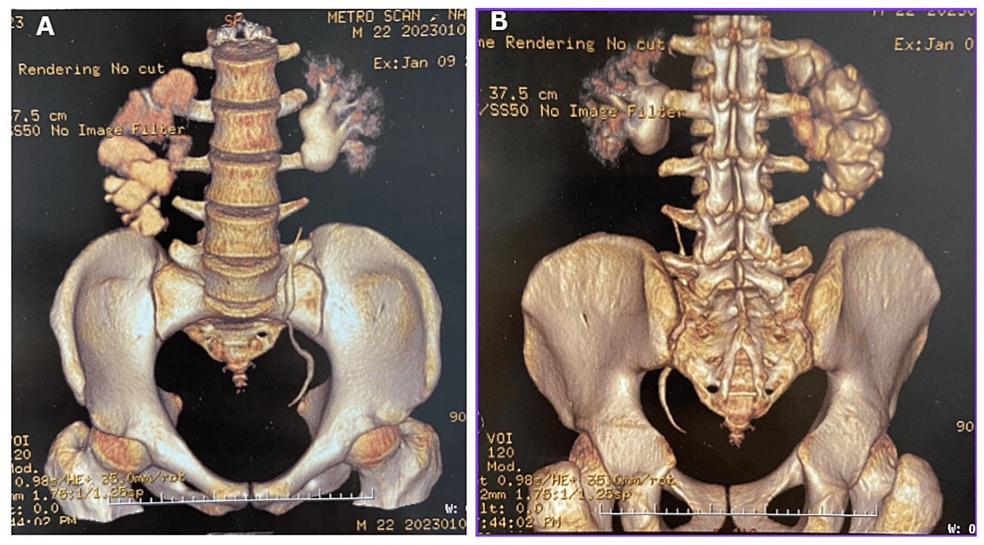
CT scan: anterior and posterior views of the right kidney CT: computed tomography

**Figure 2:**
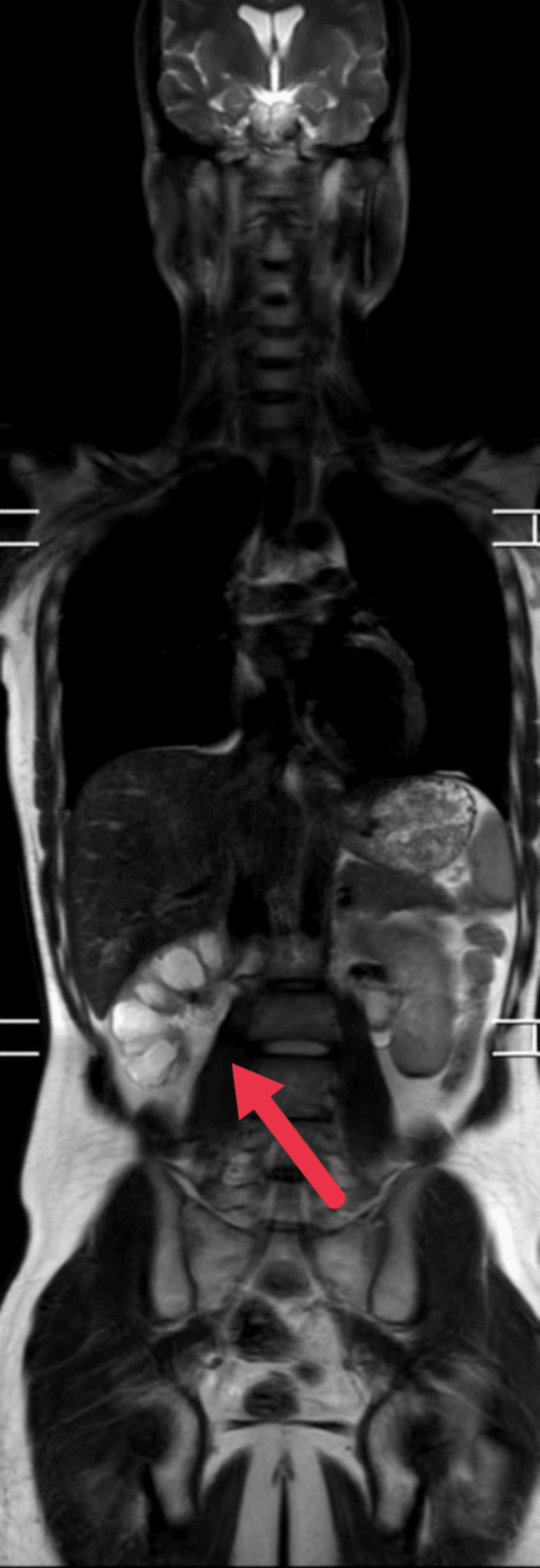
MRI showing grade 5 hydronephrosis MRI: magnetic resonance imaging

Preoperative USG of the abdominal cavity and pelvic organs performed for the first time on December 30, 2022, revealed severe hydronephrosis of the right kidney with thinning of the renal parenchyma. No obvious intestinal abnormalities were found. A DTPA renal scan (renal scintigraphy) was then performed, which revealed moderate parenchymal dysfunction in the right kidney with mildly enlarged hydronephrosis and an obstructive pattern of delayed secretion at the right iliac-ureteric junction. The total glomerular filtration rate was 78.6 ml/min (right kidney: 31.9% and left kidney: 68.1%). CT with IV contrast revealed moderate right-sided hydronephrosis, possibly due to obstruction of the pelvic-ureteral junction. On the right, it was accompanied by thinning of the parenchymal tissue. The diameter of the right hip joint was 3.3 cm (Figures 1A, 1B). MRI showed grade 5 hydronephrosis with a plaque-like cortex in the right kidney (Figure 2).

Diagnosis 

Based on the aforementioned findings, the patient was diagnosed with gross hydronephrosis due to PUJ obstruction in the right kidney and underwent laparoscopy-open pyeloplasty.

Therapeutic interventions

**Figure 3:**
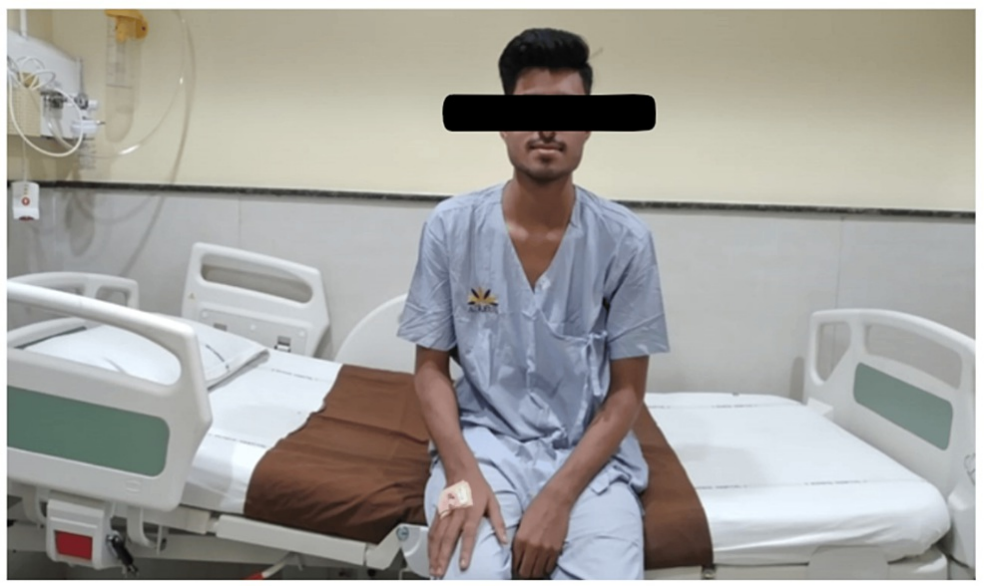
Bedside sitting position of the patient

The major goal of physical therapy for this patient was to assist in achieving a seamless transition back to his routine everyday activities, with weariness and shortness of breath being prioritized. Recognizing the patient's goals, our therapy objectives included a diverse approach. We combined traditional physical treatment with the novel use of VRT to improve the recovery process. This cutting-edge technology aims not only to alleviate the patient's dyspnea, reduce discomfort, and enhance breathing but also to encourage relaxation and improve the overall functional condition. Figure 3 depicts the patient's remarkable journey, in which he underwent a full physical treatment program while also exploring the immersive world of VRT over the course of four weeks.

Physiotherapy treatment with virtual reality therapy

VR apps for abdominal surgery rehabilitation are helpful tools that benefit patients in their postoperative recovery and rehabilitation procedures after abdominal operations. These programs provide patients with an immersive and engaging environment that can encourage and support them in recovering mobility, strength, and general well-being. The physiotherapy intervention is divided into two phases. Phase 1 involved interventions in week one (Table 1). Treatment was continued from week two to week four with additional interventions (Table 2).

**Table 1:** Interventions provided in week 1

Sr. no.	Goals of physical therapy	Therapeutic interventions	Treatment plan
1.	Prevention of pulmonary, circulatory, and tissue problems after surgery	Manual positioning: initially, a semi-reclined position was recommended. Then, the patient was made to stand up straight. Air mattresses and an ankle pump were provided	Positions were initially performed every 2 hours, 10 times x 1 set, 2 times a day, then 10 times x 2 sets, 3-4 times a day
2.	Contributes to the cleaning of the respiratory tract	Percussion and vibration of the chest and the hand	(1) Initially for 1-3 days; (2) 4-8 days
3.	Improving bed mobility and avoiding prolonged immobilization	Transition exercises and maneuvers in bed were provided with a binder, and the patient was monitored for side-turning in bed, lying down, sitting, standing, and walking	Days 1-3: rolling, prone; days 4-8: sitting in a supported position; day 9+: the Nowruz Sneer
4.	To prevent stress on the incision and suture site	Abdominal binders	Binder support during movements
5.	To improve breathing, slow down breathing, and increase breathing rate	Deep breathing exercises include (1) diaphragmatic breathing, (2) segmental breathing, and VR applications	Initially, 10 repetitions x 1 set twice a day; thereafter, 10 repetitions x 2 sets 3-4 times a day. Guided breathing exercises and meditation sessions are frequently included in VR apps
6.	Prevents joint stiffness by maintaining joint integrity and mobility	Bilateral upper and lower leg AROM exercises	Initially, 10 repetitions x 1 set twice a day; thereafter, 10 repetitions x 2 sets 3-4 times a day
7.	Back to normal ADL	Walk freely along the 30-meter corridor	On the 4th day after surgery, start with 5 minutes and gradually increase to 15-20 minutes

**Table 2:** Interventions provided in weeks 2-4

Sr. no.	Goals of physical therapy	Therapeutic interventions	Treatment regimen
1	Contributes to the cleaning of the respiratory tract	Active cycle of breathing technique (ACBT)	8th day onwards
2	To avoid muscle atrophy and to preserve muscular strength and endurance	Bridging, knee rolling, therapy band strengthening, and weighted cuff exercises are examples of upper and lower leg strengthening exercises	One week after surgery, start with 1 set of 10 repetitions twice a day
3	To improve core stability	Pelvic tilt and gentle abdominal contractions	1 set of repetitions 2 or 3 times a day
4	To reduce stiffness and pain and improve range of motion	Manual therapy, massage, and stretching	10-15 minutes of massage and stretching, 2 or 3 times a day
5	To break scars and improve tissue healing	Instrument-assisted soft tissue mobilization tools	Frequency of 1-2 times per week with a range of 5-15 minutes per area
6	To reduce anxiety and promote relaxation	Technology-assisted therapy using virtual reality therapy	20-30 minutes a day

Abdominal surgery is a complicated and intrusive treatment that can cause patients great pain, anguish, and worry. Postoperative problems are prevalent after a major abdominal surgery, with one-third to half of all patients experiencing some form of complication [8]. VRT offers a noninvasive, drug-free approach to post-surgery pain management and anxiety. By donning a VR headset, patients enter a three-dimensional world tailored to their preferences, featuring calming visuals, soothing music, and interactive elements [9]. This immersive experience enables patients to direct their focus away from surgical discomfort, promoting a smoother recovery process. The details of the follow-up and monitoring of results are presented in Table 3.

**Table 3:** Outcome measures related to the study NPRS: numerical pain rating scale; IS: incentive spirometer (in cubic inches); FIM: functional independence measure

Outcome measure	Pre-treatment scores	Post-treatment scores
NPRS	9	3
IS	0.8	2
FIM	3	7

## Discussion

Chest physiotherapy after abdominal surgery is generally provided to prevent or reduce complications such as impaired pulmonary function, atelectasis, pneumonia, and sputum retention [10]. This physiotherapy procedure involves techniques such as early mobilization, positioning, breathing exercises, splinted coughing or huffing, percussion, vibration, active cycle of breathing techniques (ACBT), as well as the use of various mechanical devices [11]. Another advantage of physical therapy is that it helps with coughing or breathing. All abdominal surgery patients should resume walking as soon as possible, as prolonged bed rest may have adverse consequences [4].

VR-assisted techniques can help people feel less worried and provide a sense of well-being following abdominal surgery. The patient may feel anxiety and sadness following surgery, which may impede their recovery and quality of life [8]. VRT has shown potential as a method for enhancing physiotherapy efficacy in patients undergoing abdominal surgery. VRT employs a computer-generated milieu to give patients immersive experiences that can improve their participation in physiotherapy activities and aid their recovery. This case study focuses on efforts to prevent or resolve postoperative pulmonary complications (PPC), thereby enabling patients to recover faster. Physical therapy helps the patient return to premorbid levels. Our patient underwent physical therapy for four weeks, which had a positive effect on his condition [8]. For decades, therapists have employed VR as a technique to give virtual reality exposure treatment (VRET) in a safe and regulated manner. So far, it has not been made widely available due to cost-related issues, a lack of standardization in VR equipment and software, and a general lack of flexibility and skills required to individualize the settings for each patient [12].

## Conclusions

This case study describes a comprehensive approach to post-abdominal surgical rehabilitation, which combines a conservative rehabilitation program with the novel use of VR treatment. The patient underwent four weeks of hard physical treatment as part of the rehabilitation program, during which the complementary use of VRT played a critical role in enhancing the entire healing process. The VRT sessions were consciously developed to target specific therapeutic goals. These goals included not only improving the patient's breathing pattern, but also major advancements in functional ability, a substantial decrease in pain levels, and significant improvements in chest mobility and activities of daily living. VRT's immersive and interactive nature provided the patient with a new and compelling avenue for attaining these rehabilitation goals, eventually leading to the patient's full recovery and enhanced quality of life.
